# Identifying individuals with intellectual disability who access mental health support and are at high risk for adverse clinical outcomes: cohort study

**DOI:** 10.1192/bjo.2023.574

**Published:** 2023-10-10

**Authors:** R. Asaad Baksh, Rory Sheehan, Angela Hassiotis, James Smith, Andre Strydom

**Affiliations:** Institute of Psychiatry, Psychology, and Neuroscience, King's College London, UK; and The LonDowns Consortium, London, UK; Institute of Psychiatry, Psychology, and Neuroscience, King's College London, UK; Division of Psychiatry, University College London, UK; and Camden Learning Disabilities Service, London, UK; South London and Maudsley NHS Foundation Trust, London, UK; Institute of Psychiatry, Psychology, and Neuroscience, King's College London, London, UK; The LonDowns Consortium, London, UK; and South London and Maudsley NHS Foundation Trust, London, UK

**Keywords:** Intellectual disability, cluster analysis, adverse clinical outcomes, aggressive challenging behaviour, common mental disorders

## Abstract

**Background:**

People with intellectual disability often experience aggressive challenging behaviour and mental health issues. It can be difficult to identify those who are at higher risk of adverse clinical outcomes when in clinical care.

**Aims:**

To characterise potential subgroups in adults with intellectual disability referred to mental health services in those presenting with aggressive behaviour or common mental disorders (CMDs).

**Method:**

There were 836 adults (≥18 years) with intellectual disability and a record of aggressive challenging behaviour, and 205 patients with intellectual disability and CMDs, who were seen in specialist mental health services over a 5-year period. Cluster analysis was used to define patient characteristics associated with clinical outcome.

**Results:**

Distinct patient groups with differentiated profiles were observed in people with intellectual disability displaying aggressive challenging behaviour, and in those presenting with CMDs. Characteristics of the aggressive behaviour group who experienced adverse outcomes included being <30 years old, being male, more mentions of aggression and agitation in their clinical record, a diagnosis of pervasive developmental disorder and prescription of psychotropic medication. Characteristics of the CMD cluster that experienced adverse clinical outcomes were being older, being a White male, having a mild intellectual disability and physical health concerns.

**Conclusions:**

People with intellectual disability who experience adverse clinical outcomes can be identified with a cluster analysis approach of common features, but differ by clinical presentation. This could be used not only to stratify this clinically heterogeneous population in terms of response to interventions, but also improve precision in the development of tailored interventions.

## Intellectual disability and mental and behavioural health

Intellectual disability is a lifelong condition characterised by impairment in intellectual functioning and adaptive behaviour.^1^ Intellectual disability is a heterogeneous condition with multiple aetiologies and exists across a continuum of severity, affecting approximately 1% of the population.^2^ People with intellectual disability are at higher risk of developing co-occurring mental illness,^3^ and around a fifth display behaviour that challenges.^4^

Mental health issues such as common mental disorders (CMDs), meaning depression and/or neurotic and stress-related disorders, are more frequent in people with intellectual disability and more persistent than in the general population.^5^ Behavioural concerns, such as aggressive challenging behaviour in people with intellectual disability, are another common reason for referral to specialist services,^6,7^ and can result in a range of adverse outcomes, including direct harm, exclusion from services and an increased risk of restrictive interventions such as psychotropic medication prescription and physical restraint,^8,9^ as well as often becoming a longstanding problem.^10,11^ Management of both CMD and aggressive challenging behaviour are compounded by people with intellectual disability being more sensitive to side-effects of psychotropic medication,^12^ which may contribute to adverse clinical outcomes and increased utilisation of services and costs.^13^

## Research using routinely-collected clinical data

When people with intellectual disability are referred to mental health services in the UK, a substantial amount of data are collected during their care, including demographic characteristics, diagnoses and clinical variables, and data on service use and outcomes such as hospital admission. The routine use of electronic health records in mental healthcare, and the rich clinical data-sets that are generated, enable statistical approaches that are not possible using traditional methods.^14^ Applying machine learning techniques to large clinical data-sets provides an opportunity to explore data in novel ways and generate new insights.^15^ One such technique is cluster analysis, which is the method of delineating distinct subgroups in a data-set such that the features of one group are more similar to each other than the features of another group.^16^ Clustering has been used to identify clinically meaningful groupings of patients within heterogeneous populations, including people in intensive care,^17^ people with diabetes,^18^ people with psychosis^5^ and people with autism.^19^ Findings from studies using cluster analysis can have important implications for the provision of care, by stratifying risk and providing targeted interventions for the most at-risk groups.^20^

Despite the potential benefits of the approach, cluster analysis is currently underutilised in research on people with intellectual disability who present to services with behavioural or mental health concerns. It is likely that improving the identification of people with intellectual disability who may benefit from more intensive interventions might reduce adverse clinical outcomes regularly experienced by people with intellectual disability, and provide the foundations for a more personalised approach to treatment pathways.

The aim of this study was to use a data-driven approach to identify and characterise clinical subgroups of adults with intellectual disability who present to mental health services that are associated with adverse outcomes. We hypothesised that patients with intellectual disability presenting with aggressive challenging behaviour or who have a CMD diagnosis may experience adverse outcomes, and these individuals would exhibit distinct clinical profiles.

## Method

### Study design and setting

This was a retrospective cohort study using data from the South London and Maudsley (SLaM) National Health Service (NHS) Foundation Trust in the UK. SLaM provides mental healthcare to approximately 1.3 million residents in four diverse South London boroughs. The Clinical Record Interactive Search (CRIS) system, developed by the Biomedical Research Centre at Guys and St Thomas NHS Foundation Trust and King's College London,^21^ was used to access de-identified structured and open-text data held in the electronic clinical records used by all SLaM services. CRIS can also provide additional data on healthcare activity that occurs in settings beyond SLaM, through linkage with other data sources such as Hospital Episode Statistics (HES)^21,22^ provided by NHS Digital. HES is a data-set curated by the NHS that includes data on all admissions, out-patient appointments and emergency department attendances at NHS hospitals in England.^23^

### Participants

Individuals included in the study were 18 years old or older at cohort entry, and had a documented diagnosis of intellectual disability according to ICD-10 diagnostic criteria (codes F70–F79).^1^ Participants must have had an episode of out-patient care including direct contact with a specialist community intellectual disability mental health team within SLaM between 1 January 2014 and 31 December 2018. Very brief episodes of care recorded as lasting <14 days were excluded on the basis that such episodes were likely to include only single assessments or to be inappropriate referrals to the specialist team that were quickly closed. Episodes of care may have continued after the cut-off date, but no data were included beyond 31 December 2018.

We included two groups of individuals taken from a larger sample of 1225 patients (477 women and 748 men):^6^ (a) 836 (68.2%) people with intellectual disability with aggressive challenging behaviour (median age 34 years) and (b) 205 (16.7%) people with intellectual disability and a CMD diagnosis (median age 40 years). Aggressive challenging behaviour included mentions of aggression within patients’ clinical notes,^6^ and CMD was defined as a diagnosis of depression and/or neurotic and stress-related disorders.

### Data collection

Clinical information within the SLaM clinical records system is recorded in either structured data fields (e.g., numerical data or data points chosen from a predefined list) or unstructured fields that contain free text written by clinicians, which may detail clinical observations and treatment plans. Diagnoses are recorded with diagnostic codes based on the ICD-10.^1^ Data in the unstructured fields were extracted with natural language processing (NLP) applications developed with General Architecture for Text Engineering software for Windows (University of Sheffield; see https://gate.ac.uk). The NLP algorithms search through the free-text fields of clinical documentation by using a machine learning approach to extract relevant information.^21^ The specification and performance of the NLP algorithms can be found in an online library.^24^ Data from three groups of NLP applications were used in the present study: symptoms/behaviours, interventions and medications.

### Variables and data sources

#### Clinical diagnoses

The following clinical diagnoses were extracted from structured fields: non-affective psychotic disorders (F2*), bipolar affective disorder/mania (F30–F31), depression and other mood disorders (F32–F39), neurotic and stress-related disorders (F4*) and pervasive developmental disorders (PDDs; F84*). We combined non-affective psychotic disorders and bipolar disorder/mania diagnoses into a severe mental illness (SMI) category. Diagnoses of selected medical comorbidities were also extracted: epilepsy (G40*), metabolic diseases (E70–E90), and congenital and chromosomal disorders (Q00–Q99).

#### Other clinical features

NLP applications were used to extract mentions of verbal or physical aggression or agitation from the free text. Examples of free text in which patients were seen to be exhibiting aggression include ‘physical aggression – in this category of behaviour, XXXXX has the potential to hit staff. She may threaten to do so to begin with by waving and shaking her fist' and ‘In [month] [year], in the context of asking staff for money, XXXXX became aggressive and is reported to have shouted and threatened staff and caused damage to property in the home. The police were called'. Agitation was included in light of evidence to suggest that it is on the pathway leading to aggressive challenging behaviour,^25^ and our previous work has shown that it is an important factor in predicting aggressive challenging behaviour and adverse clinical outcomes.^6^ Entries in the medical record that mentioned the need for social care support (defined as ‘instances of receiving current, recommended or planned general care package …  a generic term relating to any social care intervention’) or home care (defined as ‘instances of home care/help, that is, help by someone who comes to assist the patient with activities of daily living’) were also extracted; increased mentions of social care support or home care in the clinical notes often relate to concerns with the level of support, or imply that changes are being considered or made to the support package.^6,24^

#### Medication

Use of any psychotropic medication was extracted by NLP applications. Medication was categorised according to British National Formulary subchapters: hypnotics/anxiolytics, antipsychotics, antidepressants, attention-deficit hyperactivity disorder medication (section 4.4) and antiepileptics. Thyroid medication, analgesics and laxatives were combined into one category, as prescriptions for common physical health conditions.

#### Clinical outcome

Data acquired by data linkage to HES for each patient was used to create an adverse clinical outcome variable. For the purposes of the present study, this outcome was defined as one or more of the following: (a) admission to a mental health hospital, (b) admission under the Mental Health Act 1983, (c) contact with a mental health crisis team and (d) attendance at an emergency department.

### Patient and public involvement

Patient and public involvement was provided via the Personalised Treatment Packages for Adults with Learning Disabilities Who Display Aggression in Community Settings (PETAL) programme (https://www.ucl.ac.uk/psychiatry/research/epidemiology-and-applied-clinical-research-department/petal-programme). The work reported here was discussed with experts by experience at pre-application stage, and the research team and the experts-by-experience advisory group have engaged in regular updates about the project. Research using data obtained from the CRIS system is subject to approval from the CRIS Oversight Committee, which is patient led.

### Bias and missing data

Data were collected from the electronic health records of patients referred to SLaM clinical services. Therefore, only people seen by the service were included, which may have biased patient enrolment as it is possible that not all individuals who displayed aggressive challenging behaviour or mental illness were referred. Missing data owing to incomplete records were treated as missing in all analyses.

### Data access and linkage

The authors assert that all procedures contributing to this work comply with the ethical standards of the relevant national and institutional committees on human experimentation and with the Helsinki Declaration of 1975, as revised in 2008. Access to the data was granted by the CRIS Oversight Committee in accordance with CRIS's overarching ethical approvals for research use of extracted clinical data (Oxfordshire Research Ethics Committee C, approval number 18/SC/0372). Data linkage between SLaM and HES data-sets was conducted by the CRIS team in a secure environment. We did not have direct access to any identifiable data or the population used to create the study cohort.

### Statistical analysis

Distinct subgroups of patients with intellectual disability who display aggressive challenging behaviour were identified with a clustering approach, using the Gower distance, partitioning around medoids (PAM) algorithm and silhouette plot method from the cluster package in R. Data used in this analysis included demographic information about patients collected by SLaM services and the clinical variables described above. First, the Gower distance was used to calculate how similar or dissimilar individuals were to each other.^26^ This was the most appropriate metric because our data consisted of both numerical and categorical variables. The continuous variables were log-transformed because of skewness in their distribution before the Gower calculations were completed. Once our distance matrix was created, a combination of the silhouette plot method, which measures a combination of intracluster homogeneity and intercluster heterogeneity of the clusters,^27^ and judgements on the clinical meaningfulness of the number of clusters were used to decide how to partition the data. Finally, the PAM clustering algorithm was used to create the clusters.^28^ The PAM algorithm split the data into a predetermined number of clusters and chose one person with intellectual disability (an exemplar) who best represented the cluster they belonged to. Software R version 4.1.3 for Mac (R Foundation for Statistical Computing; see https://www.R-project.org/)^29^ was used for all data analyses.

## Results

### Description of the study population and clinical profile

As Table 1 shows, those with aggressive challenging behaviour spent a median of 14 months under the care of a specialist community intellectual disability mental health team. They were predominantly White (54%) and male (65%), and 38% of those with a known degree of intellectual disability had mild intellectual disability. In terms of clinical diagnoses, 45% of people were diagnosed with a PDD, 19% had a CMD diagnosis and 16% had an SMI diagnosis. Over a third (36%) had mentions of having a social care package and/or home care package in their clinical notes, and there was a median of zero prescriptions of physical health medication (as defined by this study) and one prescription of psychotropic medication. Aggressive challenging behaviour was more common than agitation (four and two mentions, respectively), and the median number of adverse clinical outcomes was zero.

**Table 1:** Demographic and clinical information of people with intellectual disability

		Aggressive challenging behaviour, *n* (%)	CMD diagnosis, *n* (%)
Total sample		836	205
Median age in years (IQR)		34 (26–54)	40 (29–55)
Gender		295 (35.3)	97 (47.3)
Female Male		541 (64.7)	108 (52.7)
Intellectual disability level Mild Moderate Severe/profound Not specified/unknown		314 (37.6)	122 (59.5)
		204 (24.4)	30 (14.6)
		120 (14.4)	13 (6.3)
		198 (23.7)	40 (19.5)
Ethnicity White Black Asian and other ethnicities		447 (53.5)	126 (61.5)
		265 (31.7)	44 (21.5)
		124 (14.8)	35 (17.1)
Median episode length in days (IQR)		417 (191.75–743)	292 (104–701)
PDD diagnosis		375 (44.9)	74 (36.1)
Ever diagnosed with CMD		157 (18.8)	–
Ever diagnosed with SMI		134 (16.0)	30 (14.6)
Ever diagnosed with epilepsy		96 (11.5)	24 (11.7)
Genetic, metabolic or chromosomal disorders		97 (11.6)	19 (9.3)
Median number of psychotropic medications		1 (0–2)	1 (0–2)
Median number of physical health medications		0 (0–1)	0 (0–1)
Mention of social care package and/or home care package		297 (35.5)	51 (24.9)
Median mentions of aggression (IQR)		4 (2–9)	2 (0–8)
Median mentions of agitation (IQR)		2 (0–6)	1 (0–4)
Median number of adverse outcomes (IQR)		0 (0–1)	1 (0–1)

CMD, common mental disorder (depression and/or neurotic and stress-related disorders); IQR, interquartile range; PDD, pervasive developmental disorder; SMI, severe mental illness (non-affective psychotic disorders and/or bipolar disorder/mania).

People with intellectual disability and a CMD diagnosis spent a median of 9.5 months being seen by a specialist community intellectual disability mental health team. Approximately 60% were White, 53% were male and 60% of those with a known degree of intellectual disability had mild intellectual disability. A total of 36% had a PDD diagnosis and approximately 15% had an SMI diagnosis. A quarter of patients had mentions of having a social care package and/or home care package in their clinical notes, and there was a median of one prescription of psychotropic medication and zero prescriptions of physical health medication. Finally, there was a median of two mentions of aggressive challenging behaviour, one mention of agitation and one adverse outcome in this group.

### Clusters of people with intellectual disability who display aggressive challenging behaviour or had a CMD diagnosis

**Fig. 1:**
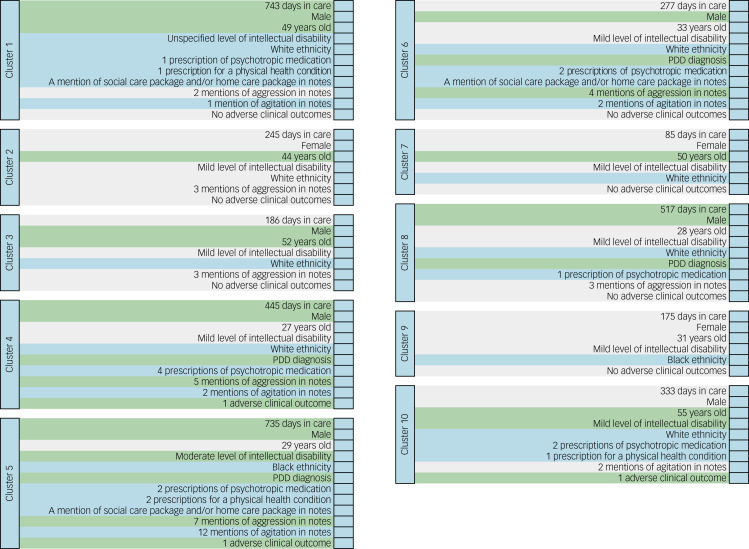
Exemplars for each cluster of people with intellectual disability who display aggressive challenging behaviour (left) and people with intellectual disability with a CMD diagnosis (right). Clusters have been colour coded to highlight key clinical features which distinguish them. Green, long episodes of care (defined here as more than 365 days in care), male gender, aged 40 years old and older, moderate level of intellectual disability, a PDD diagnosis, high levels of aggression as previously defined^6^ and adverse clinical outcomes. Grey, shorter episodes of care, female gender, aged under 40 years old, mild level of intellectual disability, low levels of aggression as previously defined^6^ and no adverse clinical outcome. CMD, common mental disorder (depression and/or neurotic and stress-related disorders); PDD, pervasive developmental disorder.

Figure 1 shows an exemplar person who best represents each cluster. The analysis yielded five clusters for those who present with aggressive challenging behaviour, with two clusters typically experiencing adverse clinical outcomes in young men, whereas the rest of the clusters were of individuals with fewer such characteristics and generally consisted of older adults. Exemplars of clusters of people with intellectual disability who had a CMD diagnosis were different compared with those found in people with aggressive challenging behaviour. People with intellectual disability in the CMD clusters predominantly had mild intellectual disability, their demographics and clinical profiles were different and only one cluster experienced adverse clinical outcomes.

### Characteristics of people with intellectual disability with adverse clinical outcomes across the groups

Two of the cluster within the aggressive challenging behaviour group were associated with adverse clinical outcomes; these were also clusters presenting with high levels of aggression (clusters 4 and 5). Those belonging to cluster 4 generally spent over a year in the service, and were on average 27-year-old men with a PDD diagnosis, of White ethnicity and with mild intellectual disability. These individuals typically had several prescriptions of psychotropic medications, displayed more aggressive challenging behaviour than agitation and experienced on average one indicator of adverse clinical outcome. Individuals belonging to cluster 5 typically spent over 2 years in the service, were on average 29 years old, of Black ethnicity and with moderate intellectual disability. They typically had a co-occurring PDD, a median of two prescriptions of psychotropic medications, two prescriptions for physical health conditions and often a mention of social care package and/or home care package in their clinical notes. On average, these individuals had seven mentions of aggressive challenging behaviour as well as many mentions of agitation in their notes, and experienced at least one indicator of adverse clinical outcome.

Of note, clusters with adverse outcome in individuals with aggressive challenging behaviour tended to include young men with co-occurring PDD, and a high number of mentions of aggressive behaviour and/or agitation.

There was one cluster within the CMD group with adverse clinical outcomes (cluster 10). These individuals spent on average 11 months in care, and they were predominantly older White men with mild intellectual disability. These individuals typically had two prescriptions of psychotropic medications, a prescription for a physical health condition (in keeping with having a more complex presentation of CMD) and on average two mentions of agitation in their clinical notes.

### Other key characteristics of people with intellectual disability who experienced aggressive challenging behaviour or had a CMD diagnosis

A striking feature of clusters who did not typically experience adverse clinical outcomes was that individuals in some of these clusters had extended episodes of accessing mental healthcare (defined here as >365 days in care). People with intellectual disability belonging to cluster 1 in the aggressive challenging behaviour group spent on average over 2 years within the service. These patients were predominantly White men with an average age of 49 years, and their level of intellectual disability was mostly unspecified. Typically, individuals in this cluster had a low number of prescribed medications (both for physical and mental health reasons), but there was often a mention of social care package and/or home care package in their clinical notes. Although they displayed more aggressive than agitated behaviour, on average, they experienced few indicators of adverse clinical outcomes during their episode of care. Individuals in cluster 8 of the CMD group spent on average 17 months within the service. They were generally younger men with mild intellectual disability, with on average one prescription of psychotropic medication and a PDD diagnosis.

The clusters with the longest episodes of care (clusters 1 and 5 within the aggressive challenging behaviour group) had a mention of social and/or home care packages in the care notes, suggesting that concerns or issues in these areas may affect mental healthcare provision.

Several clusters had neither adverse clinical outcomes nor extended episodes of care. People in cluster 2 in the aggressive challenging behaviour group typically had no recorded emergency care contacts and had shorter episodes of care (on average 8 months). They were generally older White women who had mild intellectual disability and few mentions of aggressive challenging behaviour in their notes. Cluster 3 was similar to cluster 2, but individuals in this cluster were primarily men who were on average 52 years old, had short episodes of care and had generally low levels of adverse outcomes. Finally, in the CMD group, cluster 7 on average spent 3 months in care; they were generally older women with mild intellectual disability who had no indications of physical health comorbidity as indicated through medication prescription, and did not on average experience any adverse clinical outcomes.

### Clusters with individuals with PDD comorbidity

In total, four clusters were identified who typically had a PDD diagnosis. The two clusters in people who experienced aggressive challenging behaviour (clusters 4 and 5) both usually had adverse clinical outcomes. However, the two clusters with a PDD diagnosis in those with CMD (clusters 6 and 8) did not generally experience adverse outcomes. Individuals in these clusters were on average under 35 years old, male and primarily White (three out of the four clusters). Prescriptions of psychotropic medications were common in these clusters, and mentions of aggression were generally high, with the exception of cluster 8.

## Discussion

This study used real-world clinical data from specialist intellectual disability community mental health services in the UK to identify people with intellectual disability who are at risk of adverse clinical outcomes. The clusters of individuals who displayed aggressive challenging behaviour were distinct from those found in people who had a CMD diagnosis on many of the demographics, clinical characteristics and, most importantly, the clinical profile of those who experienced emergency clinical contacts and admissions as indicators of adverse clinical outcome. The findings demonstrate the utility of a clustering approach to identify clinically meaningful subgroups of patients with specific presentations.

We identified two clusters of patients with aggressive behaviour and one cluster of those with a CMD diagnosis that typically experienced adverse clinical outcomes, including being detained under the Mental Health Act. These outcomes were designated as such in view of current policy and practice recommendations in the UK that people with intellectual disability should be managed in the community wherever possible, with appropriate support.^30^ Admission to hospital may contain a situation that has become unsafe, but may not directly address the underlying cause of aggressive challenging behaviour or CMD diagnosis, which often requires longer-term interventions to be embedded in community settings. Attending the emergency department was designated an adverse outcome as this is often a difficult experience for people with intellectual disability,^31^ and may reflect missed opportunities for more timely and proactive support to avoid such acute presentations.

Although the clinical profiles of the two groups with aggressive challenging behaviour most likely to experience an adverse outcome were distinct in terms of demographic and clinical features, there were also some commonalities between them. We found that being aged <30 years, being male, having more mentions of aggression and agitation in their clinical record, having a PDD diagnosis and being prescribed psychotropic medication appear to be the shared elements in those who present with aggressive challenging behaviour and experience adverse clinical outcomes. Although previous research has attempted to identify demographic, clinical and care factors associated with aggressive challenging behaviour,^4,10,32,33^ few studies have related these to clinical outcome. Our findings suggest that services need to be better equipped to support young men with intellectual disability and co-occurring PDD; for example, by considering and addressing the needs of these young men through identification of common environmental or social triggers, and by providing assessments and adaptations targeted to autistic traits, such as sensory difficulties, or by formulating the display of aggressive behaviour through an ‘autism lens’ in addition to the classical paradigm used in people with intellectual disability. Moreover, the cluster that included adverse outcomes in those with CMD were different in terms of age, but included an indicator of physical health comorbidity, suggesting a different approach may be needed within this group to reduce the risk of adverse outcomes.

We also identified several clusters with extended episodes of care under specialist mental health services, which in two clusters had mentions of social and home care packages, suggesting a generally higher level of care needs in this group, and may also indicate deficits in social care that were a factor in the person's presentation. This could have had a negative effect on mental health management, requiring longer-term input. Care and organisational factors have been shown to be important in the development and maintenance of challenging behaviour;^34^ a range of person-centred models of care should be available, and commissioners of services should be responsive when concerns around care are raised, so as to avoid escalation and admission to hospital. In the CMD clusters, episodes of care were generally shorter than those with aggressive challenging behaviour. However, the clusters with the longest episodes of care shared similar features to the aggressive challenging behaviour clusters, including a PDD diagnosis and being male.

### Strengths and limitations

We applied a machine learning method to generate new insights from a large clinical data-set. The patient clusters that have been identified have potential to be used to stratify this clinically heterogeneous population into subgroups, and direct interventions and resources to those at greatest risk of an adverse outcome and provide more preventive care.

Data for this work were obtained from clinical services covering a socioculturally and economically diverse population of South London. We included only those who were under the care of specialist services; it is possible that not all those with mental health or behavioural concerns were referred to the service, and this may have contributed to selection bias. Nonetheless, the cohort will be representative of people with intellectual disability and aggression or CMD who access community intellectual disability services in the UK. The current findings would need to be further replicated in patient cohorts from other regions. The data were also routinely collected, which imposed limitations on the availability of variables of interest; therefore, the study could be improved by using a prospective design with more bespoke data collection. The NLP for aggression could not identify the type of aggression (such as verbal aggression or physical aggression) in patients’ clinical notes. Consequently, it is currently unclear if one type of aggressive challenging behaviour is more prevalent in adults with intellectual disability than others. There is scope for future studies to develop NLPs that are able to distinguish different types of aggression. Similarly, we did not examine the causes of aggressive challenging behaviour experienced by patients in our sample, such as level of language or somatic conditions. Future studies could investigate the potential associations between these variables and aggressive challenging behaviour, to better understand the underlying causes of aggressive challenging behaviour in people with intellectual disability. A greater number of clinical variables, and more detail of living situation and support package, could be added in future work to further refine the patient profiles associated with greatest risk. Moreover, we found relatively low numbers for prescriptions of psychotropic and physical health medications than would be typically expected in an intellectual disability sample, where polypharmacy is common.^35^ First, our medication lists were not exhaustive, and patients could have been prescribed medications not included here. The low frequency of recorded prescriptions could also suggest limitations in the recording of medication data in patient records in specialist services. Future studies may be able to provide insight into this by comparing medication records in primary and secondary care services through data linkage. A wider range of adverse outcomes, including those of most importance to patients and carers, could be added to the analysis.

In conclusion, we have demonstrated the feasibility of using a cluster analysis technique to identify unique subgroups in people with intellectual disability who present with aggressive challenging behaviour or have a CMD diagnosis, including those associated with adverse outcomes. The findings highlight the need for responsive, culturally informed and personalised interventions. The clusters identified can be used to stratify patients with intellectual disability with common presentations to mental health services according to potential outcome, and identify those who need specific treatments or more complex intervention. It could help to inform service planning, and ensure that people are managed in appropriate care pathways to increase the precision of treatment content and lead to optimisation of outcomes for this population group, as well as maximise the efficiency and effectiveness of care and prevent undesirable outcomes, such as compulsory admission to hospital.

## Data Availability

The data that support the findings of this study are available from the corresponding author, A.S., upon reasonable request.
